# Evil, Constructed: A Salient Part of an Emerging Spiritual Veteran Identity

**DOI:** 10.1177/15423050231213418

**Published:** 2023-11-09

**Authors:** Jan Grimell

**Affiliations:** 225313 Department of Sociology, Uppsala University, Uppsala, Sweden

**Keywords:** Evil, veteran, identity, existential, pastoral care, spiritual care

## Abstract

This article investigated constructions of evil among deployed Swedish veterans. Six cases were used to demonstrate common themes of these constructions: humans are capable of everything; anyone can be violated, even killed; evil and cruelty comes in many forms; coldness/cynicism; exhausting to witness suffering and pain; and existential rumination. The impact of these can affect a veteran's identity and their notions of self. However, processing encounters with evil is seen, in some Christian perspectives, as an essential prerequisite for spiritual growth, and this might be potentially important to supporting the emergence of spiritual veteran identities. A pastoral care giver or military chaplain can serve as an existential conversation partner who can assist veterans when approaching such experiences and their potential impact. This may be especially fertile in secular contexts, where pastoral wisdom and ontological approaches can be hard to find in the everyday lives of veterans.

## Introduction

There has not been a war in Swedish territory for over 200 years, giving Sweden a unique experience of peace in the Western world. In the Nordic context, neighboring countries such as Norway, Finland and Denmark have contrasting experiences due to the Second World War. A uniquely long domestic peace has implied, among other things, that Sweden has not experienced the ravages of war in its cities or countryside for a very long time. Sweden's societal culture has instead gravitated toward values, meanings, and practices—i.e., culture—which are characteristic of such a peace. A long peace inevitably creates a society in which war, evil, and suffering are viewed only from afar; except for those veterans who have been deployed to conflict and war zones elsewhere (Grimell, 2022).

Research suggests that Swedish society hosts a distinct gap between the life-world of veterans, in which deployments to conflict and war zones are an integral part of a service that can include combat tasks, death, and suffering, and a civilian life-world in which one stands on the sidelines, a good distance from conflict zones and regards conflict or war as something that happens outside and beyond ordinary life in the nation and in society. Such a gap between the veteran and the civilian has been illustrated in research at both societal and individual levels (Grimell, 2022, 2020a, 2019, 2018a, 2016). At the micro- or individual level, it can be said to create a kind of gap within the self where experiences from deployments stand in sharp contrast to the civilian life-world that also exists within the self (Grimell, 2023a). It can be complicated for veterans to engage in dialogue with an identity shaped by war zone deployment(s) and identities formed from their experiences the civilian life-world (Grimell, 2022). It can also be challenging for a veteran to share deployment experiences of evil, destruction, and violence with family, significant others, and civilians in a broader sense (Beder, 2012; Bragin, 2010; Lifton, 1992; Moore, 2012; Rumann & Hamrick, 2010).

One element that reinforces the gap between veterans and civilians is the evil and destructive forces that many veterans experience during deployment. The more a society is influenced by peace, the greater the gap becomes between society at large and veterans, and within veterans themselves due to their constructions and understandings of the evil they have experienced. The effects of such constructions can be amplified and exacerbated if, in addition, there is a lack of conceptual apparatus that offer hermeneutical keys to understanding the evil and destructiveness witnessed and experienced during deployment. A Swedish context can be said to fulfill these two premises considering the long-standing peace which widens this gap; and given that Sweden also is described as one of the most secular countries in the world (Lemos & Puga-Gonzalez, 2021), which suggest that a hermeneutical apparatus for the understanding of war-time evil is under-developed (Grimell, 2022).

The purpose of this article is to unfold an important analytical finding from a recent research project which included 24 Swedish veterans who experienced deteriorating mental health and increased suffering associated with their deployments to conflict and war zones.

The following research questions organize the article:

- How are constructions of evil described by Swedish veterans?
- How can this be understood through a pastoral care lens?

The analysis will form the basis for a conversation about the implications constructions of evil have on veteran identity and the role of pastoral care. This will be followed by a discussion on the conceptualization of evil and identity.

## Conceptualization of Evil

Concepts such as “evil” and “destructiveness” can be understood through different approaches, including those coming from sociopsychological and religious perspectives, among others. This article will conceptualize sociopsychological and religious positions as they aid in articulating two possible conceptualizations of evil.

Evil can be seen and understood through sociopsychological experiments; which was particularly well manifested in Zimbardo's (2008) book *The Lucifer effect: Understanding how good people turn evil*. Zimbardo's (in)famous sociopsychological prison experiment—the Stanford prison experiment—was based on a division of students into the category of prisoners and prison guards. Many of the students had come a long way in their education and were considered to be mature and of good moral integrity. The students were therefore divided into roles such as prison guards and prisoners, and would play these roles for a certain period of time in a closed context; i.e., it was not possible for the role holders to take a break or leave the experimental context to rest, change their environment, or leave their roles. In just a few days, the experiment derailed, as the students quickly entered their roles so deeply that they lost their previous moral compasses and made the existing imaginary world their own reality. The experiment simply had to be canceled after five days. The prison guards began to behave in a heavy-handed and brutal manner towards the prisoners, who were dehumanized and left bereft of their former identities and values. Even the prisoners were affected by the process and came to feel, and behave, like prisoners. Zimbardo called this the “power of the situation” and argued that the power of the situation can explain what, in the sociopsychological sense, can be understood as evil and destructiveness—i.e., the Lucifer effect. In the Christian tradition, Lucifer is frequently associated with Satan, an association that will unfold more closely in the next paragraph. The power of the situation should be understood in relation to a context in which supervisory functions and privacy-preserving mechanisms are either dissolved or only partially exist. This creates free rein when it comes to utilizing the power of the situation, which can then drive human behavior and actions to extreme destructiveness or evil. The view of human beings expressed in Zimbardo's research suggests that human is a pliable being, in a sociopsychological sense, who can gradually—but still quite quickly—change his behavior and moral compass if the situation and context are favorable.

Zimbardo was called as an expert witness during the legal aftermath of the infamous prison abuses in Abu Ghraib during the US invasion of Iraq (which became known in 2004). Zimbardo argued for the “power of the situation” in the absence of clear oversight and privacy-preserving functions. Based on this argument, Zimbardo argued that the burden of blame should be placed at the system level rather than on the individual. He pointed out that the power of the situation could be given free rein in the prison context in Abu Ghraib, as in the Stanford prison experiment, due to the absence of systematic mechanisms that maintained the moral stature and integrity of the military as well as the human value of prisoners.

Another understanding of evil, destructiveness, and the brokenness of humans can be found within the Christian paradigm. Such an approach often gravitates towards the Bible's accounts of the struggle between good and evil. In the oldest parts of the Bible, the Old Testament, this transcendent struggle is embodied by God and Satan—the latter a anglicization of the Hebrew word שָׂטָן meaning “opponent” or “enemy” (Bergman, 2000, p. 245). The adversary, or “Satan,” can be understood in a Christian tradition as a fallen angel who can assume different guises and walk the earth. In the Hebrew textual tradition (the Old Testament in the Christian tradition), Satan can also be understood as the “Accuser,” who is described in the book of Job as an angel subject to God. Satan, adversary, enemy, and accuser can be said to be synonyms for a transcendent agent who often subjected humans and faith to various kinds of trials and temptations in the Old Testament. In the newer parts of the Bible, the New Testament, it is the main character Jesus who continues the divine struggle against evil forces and actors such as the proposed and faith-testing Satan. In the New Testament, which was written in Greek, the name “Devil” also appears; for example, when Jesus is tempted in the desert, as described in the Gospel of Matthew. From a Christian perspective, the spiritual tug-of-war seeps down to the human level and is individualized. This can be illustrated as trials and temptations aimed at getting humans to do wrong; e.g., to not do the right thing, temptations that cause humans to gradually let go of what leads them away from what is good and edifying and into destructiveness (Girard, 2001).

In some perspectives of Christian tradition, people are also considered to have a built-in disposition or inherited brokenness that means that they have a chronic navigation problem between right and wrong that they cannot manage to sort out on their own. To this extent, human beings are weak in both the body and the spirit or soul and need spiritual and social support to manage themselves in the world in which they live (Bonhoeffer, 1959). In the wake of the spiritual tug-of-war at the human level between good and evil, destructiveness, shortcomings, atrocities, and abuses can occur in the name of evil—just as self-sacrifice, loyalty, discipline, incorruptibility, and perseverance can occur in the name of good and for a higher purpose (Girard, 2001). It should also be said that the forces of evil can either be given a more literal meaning and literal agent or understood in a mythical and metaphorical way that opens (transcendent) doors beyond the human.

A final but useful suggestion regarding the problem on evil comes from Irenaeus of Lyons. Irenaeus represents the Greek patristic thought tradition which sees human nature as a potentiality. Humans are created with capacities for growth toward maturity. But that capacity for Godward growth requires contact with and experience from good and evil if truly informed decisions are to be made. Encounters with evil are seen as an essential prerequisite for spiritual growth and development (McGrath, 2007). Irenaeus' view was further developed by Hick (1966) which emphasized that human beings are created incomplete. Growth is a process based on the free will; humans are individuals who can respond freely to God. In order to become what God intends humans to be, they must participate in the world. Unless a real choice that matters is available between good and bad, the biblical injunctions to choose to are meaningless. Thus, good and evil are necessary presences within the world, in order that informed and meaningful spiritual growth may take place. This idea is obviously attractive and relates a free will to a person's exposure to evil, potentially followed with moral action which then leads to spiritual development. But this approach also implicates that evil has an implicitly positive role to play, and that suffering can be seen simply as means of advancing spiritual growth which makes it difficult to understand events such as Auschwitz. This type of criticism is usually answered by encouraging passivity instead of action.

A working definition of evil in this article encompasses the existence of evil that is necessary–given that the world consists of the presences of good and evil–for informed and meaningful action and spiritual growth to occur. This understanding and definition is privileged over the others because veterans in general tend to change after deployments (Grimell, 2023a, 2023b; Lifton, 1992). The change revolves around new insights and deeper understandings about the conditions and complexities of life, good and evil included. The awareness of evil informs meaningful choices and actions leading to spiritual growth. This spiritual development is integrated within the veteran identity and self.

## Conceptualization of a Veteran Identity and Self

The concept of identity in this article refers to a narrative identity of “who I am.” A person can narrate a large number of storied identities; for instance, as a mum, dad, husband, wife, son, daughter, man, women, service member, veteran, football player, musician, and so on (Grimell, 2021a, 2021b). A narrative approach such as this also makes it easy to understand identity from an empirical and methodological point of view (McAdams et al., 2006). A “veteran identity,” in this article, emerges from stories of service in and deployment to conflict and war zones by the Swedish Armed Forces and other deploying agencies. The idea of the “self” equates to the person as the host of narrative identity claims. This implicates that the self maintains and navigate several potentially-contrasting narrative identities (Grimell, 2021a, 2021b, 2018a). The challenge of several characters of the self is narratively solved through the distinction between character and story. “The many are the main characters; the one is the story within which the characters are given form, function, and voice” (McAdams, 1997, p. 118).

## Method

This article draws from a study that was conducted in 2022. The study was ethically approved by the Swedish Ethical Review Authority (reference number 2021-05410-01). The purpose of the study was, among other things, to investigate deteriorating mental health among Swedish veterans through an existential lens; i.e., approaching their suffering through life questions and existential concerns. A qualitative interview method was considered particularly appropriate for the study, due to the lack of qualitative existential information from primary sources in a Swedish context. In qualitative interview research, the weight or quality of the results is not based on the number of people interviewed, although there needs to be a sufficient number of interviewees to bring out qualitative variations in the interview material. The focus is instead on creating new understandings of subjective events and phenomena that focus on people's life-worlds, experiences, and perceptions (Crotty, 1998; Polkinghorne, 2005; Verschuren & Doorewaard, 2010).

A concept such as “validity” can be useful in qualitative research (Ganzevoort, 1998; Mishler, 1991). Through a researcher's questions about, e.g., life questions and existential concerns, qualitative research can have a high validity (ability to measure what is intended to be measured) depending on how motivated interviewees are to share their experiences. Validity was fostered by asking explicit questions, organized in theme-based areas, which the study intended to explore, analyze, and develop new knowledge about (see Appendix S1).

An interview study is qualitative and understanding-driven, which means that the results of the analysis cannot be transferred to a population or group of veterans. However, this study generates new knowledge and understanding about what an existential concern might entail among veterans experiencing increased suffering. This is important, especially in a highly secularized context such as Sweden where few, if any, empirical research studies so far have highlighted the experiences of evil among veterans. To understand what experiences of evil entail and can mean, as seen through a pastoral care lens, is urgent because the perspective is missing.

### Sample

Purposeful sampling (Merriam, 2002; Polkinghorne, 2005) was done with the support of the Veterans’ Clinic at Uppsala University Hospital, and 24 veterans were interviewed in spring 2022.

The sample included veterans from both the Swedish Armed Forces and other deploying agencies. Of the 24 interviewees, nineteen were from the Swedish Armed Forces (sixteen men and three women) and five (four women and one man) were deployed by other agencies. A total of 17 men and 7 women were included. To hinder backtracking and to protect the anonymity of the interviewees, the agencies involved have not been specified (apart from the Swedish Armed Forces). Each interviewee was asked to create their own fictitious name.

**Figure 1.**
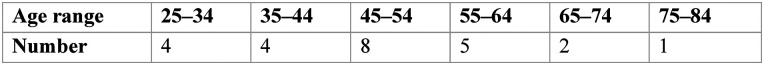
Age distribution and number of participants.

The age distribution of the interviewees was broad and is illustrated below (Figure 1).

The Army, Navy, and Air Force—the traditional branches of defense—are represented among the interviewees, although the Army and Navy form the bulk of the military participants. The interviewees illustrate a broad mix of different units from the aforementioned branches of the armed forces.

The number of overseas deployments varies widely among interviewees. Some have completed 1–2 overseas deployments, while others have completed 3–8 overseas deployments. There are also interviewees who interrupted planned or ongoing deployments for various reasons. The overseas deployments were carried out under different deployment areas ranging from the Middle East (e.g., Cyprus, Sinai, Lebanon), former Yugoslavia (e.g., Macedonia, Bosnia, Kosovo), Africa (e.g., Liberia, Mali), to Afghanistan. However, the mission focus among interviewees can be said to be centered around deployments to the Middle East and what was Yugoslavia in the 1990s, and deployments to Afghanistan and Mali in the 2010s.

The majority of participants can be described as “cultural Christians,” a Swedish concept which includes citizens who grew up in a time where the Church of Sweden was a state church and in whose rites and activities a large part of citizens participated during their childhoods, adolescence, and later in life (Kasselstrand, 2015). In addition to this, some participants described themselves as “believers” and were actively engaged in their Christian religion.

### Interview Design

The interview phase started during an intense wave of COVID-19 infection in Sweden in early 2022. The majority of the interviews were therefore conducted via videoconference. A number of interviews were conducted by mobile phone due to poor internet connections, technical problems, or a lack of digital technology that enabled interaction via videoconferencing. Before the interviews were conducted, informed consent had been signed and sent to the researcher. There was also an opportunity to ask questions before the interview started.

The interviews were based on a semi-structured interview design which was formalized in an interview guide containing twenty-five themed interview questions that addressed a number of sub-questions regarding areas like existentialism (see Appendix S1 for a full disclosure of interview questions). These questions were broad, open-ended, and descriptive in nature, and had no simple answers. For example, the interview questions were formulated as follows:

- Are there any questions about your life that have arisen in light of your well-being?
- In what way have your deployment experiences influenced your outlook on life?
- What questions about life or life issues do you carry with you today?

The purpose of open-ended interview questions was to encourage interviewees to tell their own story and share their experiences (Clandinin & Connelly, 2000; Mishler, 1986, 2004). The open-ended question methodology creates a good opportunity for interviewees to respond to questions in their own words (Webster & Mertova, 2007).

The total interview time, all interviewees included, was 29 h and thirty minutes.

### Analysis

The researcher conducted the analysis through the following phase-based process.

The first analytical step was to manually summarize the interview narrative in a notebook after each interview. In this process, the interview was condensed down to a core narrative of one or more pages. Approximately 1½ notebooks were used in this handwritten process. This type of interview summarization into a core (life) narrative can be called a “global reading,” and is an approach in narrative analysis (Ganzevoort, 1998). The aim of this type of global analysis is to gain an overview of key findings and contours in the interview narrative.

The next steps were transcription, close reading, and coding of the interview material; which involves the researcher delving more systematically into the interview data. In order to be able to manage all the interview transcripts, keep track of all the interview codes in an easily-manageable way, and sort them into different groups, a qualitative analysis software called Atlas.ti was used.

Inductive logic was used during the analysis process, which involved a movement from many individual small codes to general overall themes. The process is based on coding important and interesting individual findings in the interview material and then developing the observations into general themes or group-level categories called code families in Atlas.ti. Hypotheses and theory can be constructed and developed using the information at the group level (Kvale & Brinkmann, 2009).

Not all individual codes are automatically reflected in a code family. However, the associations of a code should lead to the code family, and vice versa. Some of the individual nuance is lost in the movement from the individual to the general. At the same time, in an inductive process, individual coding needs to be sorted in some kind of way in order to create overview and order; otherwise it becomes difficult to describe the results of the analysis in a meaningful way. All individual codes were organized into thirteen code “families,” and code family code seven—which covered existential doubts and life issues/views—is unpacked in this article. The full result of the analysis has been presented in a book only available in Swedish (Grimell, 2023b).

Six representative cases have been selected for the results section, which together illustrate various constructions of evil that were present among the interviewees. In the presentation of these cases, grades, positions, specific deployments, and other detailed information, including age, have been omitted to hinder backtracking.

## Results

The interviewees’ experiences from their deployments to conflict and war zones had chiseled out a salient narrative about evil and destructiveness. Experiences from deployments had established a rather harsh or crass view of the nature of humans, as well as of life itself. The implication of these experiences can be understood as a kind of wisdom around evil, or the dark, destructive and life-destroying forces that were an inescapable element of the deployments to conflict and war zones, and are inescapable elements of life itself. The ontology of evil was not explicitly addressed and discussed during the interviews, which is why the ontological question remains relatively open to the approaches presented earlier in the article. Independent of the ontology, the awareness and understanding of the human complexity and the existing destructiveness were often referred to as “evil”; which was clearly articulated as a concept among the interviewees.

David was close to 50 years of age and an officer experienced in combat who had served for decades until he decided to finally transition from active duty to a civilian life. David's encounter of evil actions and events during his missions overseas had changed his perception of the character of humans and his view of humanity. David explained his insights in this way:

I don’t know where to start, but man is a rather egotistical character, and has his own goals and has his own purposes. A pretty evil creature also in many respects, if you look at what happened down there [in the context of the military operation] and everywhere in the world today. Then again, I can’t say I go and think about it constantly, because then one wouldn’t have been able to live. But it’s probably some kind of…I can’t say I’m a realist, I never have been, but I got crasser after those deployment experiences down there. Cold maybe, a little colder, crasser, a little more cynical somehow. […] If you look at people and the world, then it gets a little darker actually. What are we doing to each other? It's crazy.

Not only did David's experience of evil actions change his perception of humans, they also reshaped his identity as a veteran by adding an element of coldness and cynicism. Another interviewee, Patrik, talked about how war brought out the worst and best in humans. Patrik was over 50 years old, and had been deployed numerous times to conflict and war zones between the ages of 20 and 30 before he transitioned from military to civilian life. Patrik had seen the worst as well as the best things in humans amid deployments. His conclusion was that human beings—including himself and friends—at their very core, had a changeable character. Patrik described:

I say this to friends and acquaintances like this, that “war brings out the absolute worst in man and at the same time the best”. Life is a grayscale, it is not black or white and if we end up in similar situations, we can also change drastically. Unfortunately, this has been reminded of in the Ukraine war. […] It is always women and children who are hardest hit.

Patrik's experiences also highlighted that the vulnerable, i.e., “women and children,” are also those who are most vulnerable and suffer the most.

Harald, another veteran between 50 and 60 years old, shared Patrik's experience about the duplicity or changeability of humans. Harald had been deployed numerous times by other deploying agencies. He had witness and experienced the human suffering emerging from conflict and war zones across the globe. Harald talked very explicitly about good, evil, and about the complexity of man. Harald had ruminated extensively about these topics over the years. With a sigh, he started to share his insights on the topic:

[Sighs] Yes, it is so multifaceted. Man is such a complex creature. We can do so much good, and we can do so much evil. I’ve seen such amazing things that I wouldn’t have gotten done without my overseas assignments—and the most horrible, horrible thing you can think of. And what is it that drives us to do one or the other, it’s incredibly difficult to know. And I don’t have any answers to that, but I just note that it is so, and I think a lot back and forth without coming to any real answers. Except that we humans are so complex. One second, we can do something incredibly good and the next second the same individual can do something incredibly evil. There are no evil [people], there are no good ones. There are only people who are complex. But how you can go one day from doing something incredibly good to doing something incredibly evil the next day, I don’t understand that, that process. I understand that it is happening, and I understand somewhere how it can be done, but not really why the barriers are not there.

Many interviewees had directly or indirectly witnessed abuse, violence, ethnic cleansing, misery, and suffering in various conflict and war zones from the 1970s until today, and these impressions had shaped their views of human beings, humanity, and of life itself.

Interviewees had first-hand experience with the idea that a human life in a conflict or a war context had neither a particularly high value nor was it inviolable. It could be violated in every possible conceivable and unthinkable way. It could be enough that the neighboring family one had lived next to for a lifetime suddenly had the wrong ethnic origin in a conflict. Such an ethnic difference could legitimatize the killing of the whole family. The veteran Loffe had been deployed by the Swedish Armed Forces during the 1970s. He was the oldest interviewee; close to 80 years old. Loffe pointed to the fact that everything can be violated in conflicts and war-time—nothing is sacred. He retold the incident of a massacre based on ethnicity in a specific area in the deployment context in which he served:

There were massacres in a couple of villages that were ethnically mixed. And it was battles and combat going on and [one ethnical group] got cold feet. They understood that “we can’t stay here”. So, they took care of [the other ethnic group] in the same village. They had lived there for hundreds of years. ‘Took care of’ them; well, it was mostly babies, women, and older men. The younger men were out in the combat field fighting. Before this ethnic group escaped, they shot them all with neck shots. And then they hid them in the village garbage heap. They buried them there. Don’t know, think it was about [a three-digit number of murdered people] in a village. And then they took off, they escaped. So, it was a massacre. Then [people from the other ethnic group] came to the village and when they got there, they looked everywhere. The village was empty. Where are all the relatives, all of them? So, they started to search and found them in the village garbage heaps. Whereupon then, as usual when it is one side or the other, but here it was justified, they complained to the UN. […] Yes, things like this happened.

Experiences of evil, destructiveness, and violence—which were difficult for Loffe to grasp and handle, and would be for any veteran—prompted different types of existential thought-complexes among the interviewees. Such implications of deployments needed to be dealt with and interpreted by veterans in relation to their life situation beyond service. This process could be understood as a type of existential rumination, which stood in particular contrast—even becoming surreal—as veterans encountered everyday life in Sweden. On a social level, this frequently gave rise to frustration, irritation, or moral indignation over the things which civilians in the Swedish peace-society complained about or criticized. On a personal level, these existential ruminations were both exhausting and difficult to bring to a close.

Erika testified to the existential challenges she experienced on a personal level. She was a veteran in her mid-thirties and had a military background, but had been deployed numerous times by other deploying agencies. Constantly witnessing human suffering and seeing destructive forces tear down all work which the international community did were contributing factors to her sick-leave. Erika recounted:

I had worked very hard, both when the conflict in [an international hot spot] started and then with a very shaky situation in [another conflict area] just months before. So, I also think I was pretty exhausted in terms of witnessing human suffering and the feeling of doing everything one can yet everything falls apart anyway. […] It became very strong for a couple of months, and I was on sick-leave.

LOLULL^1^ sums up the existential challenges on both a personal and a social level. He was a very experienced veteran between 60 and 70 and had been deployed across the globe numerous times by the armed forces and other deploying agencies. He had also lost close colleagues during deployment. LOLULL recounted:

I was at the field hospital [in a conflict area], where children who were genitally mutilated were admitted. They had thrown Molotov cocktails into the schoolyard, so we had burned children, as well. This was particularly excessive violence. So, in an armed conflict, I think I can accept that in an armed conflict you get hurt, you can even be killed. But this excessive violence that I saw in [a conflict zone]. I’ve seen it in [another conflict zone] where they walked around with machetes. I saw it in [a third conflict zone]. Those feelings, there’s an awful lot of aggressiveness. […] It’s obvious that I’m sad that man can be so cruel. […] So, this cruelty, I think one gives the other. Man always finds a reason to take revenge, and so it begins. That's how it escalates. I guess that’s the explanation I’m trying to console myself with. But I can’t find a really good answer to this. I probably have to reflect a little bit more. I must mention, I have given up on telling others at home what I have been through. No one is interested.

## Discussion

Veterans have seen and experienced events, conditions and life stories that, as a rule, civilian people in a society at peace have not experienced (Beder, 2012; Bragin, 2010; Lifton, 1992; Moore, 2012; Rumann & Hamrick, 2010). This can, of course, increase the perceived gap between veterans and civilians; especially in a nation where the peace has lasted for more than two hundred years (Grimell, 2022). This is a common motive or theme among veterans for not sharing their experiences of evil with family, friends, or the wider social network of civilians. But at the same time, this means that veterans carry a kind of burdensome wisdom about life that is unique and colored by their deployments. This can be understood as a strength, and a reminder that evil and destructiveness is not especially far away from everyday life. The perception of distance from evil is just that—a perception. In fact, the volatile nature of evil makes life very fragile and vulnerable. Life's so-called inviolability and human rights can in fact be renegotiated and changed quite quickly and may depend upon whether one lives on the right side of a border, or has the right ethnicity or kinship.

More importantly, as seen through the pastoral care lens in this article, the veterans’ encounters with evil serve as the prerequisite for spiritual growth and development (Hick, 1966). This suggests the veterans, due to the exposure of, and experience from, evil amid deployments, have been forced to make real decisions and act to protect and support civilians, themselves, and others. In line with the Greek patristic tradition of thought (McGrath, 2007), the only way to uncover the potentiality and develop spiritual growth is to participate in the world where real choices must be made, and actions performed.

**Figure 2.**
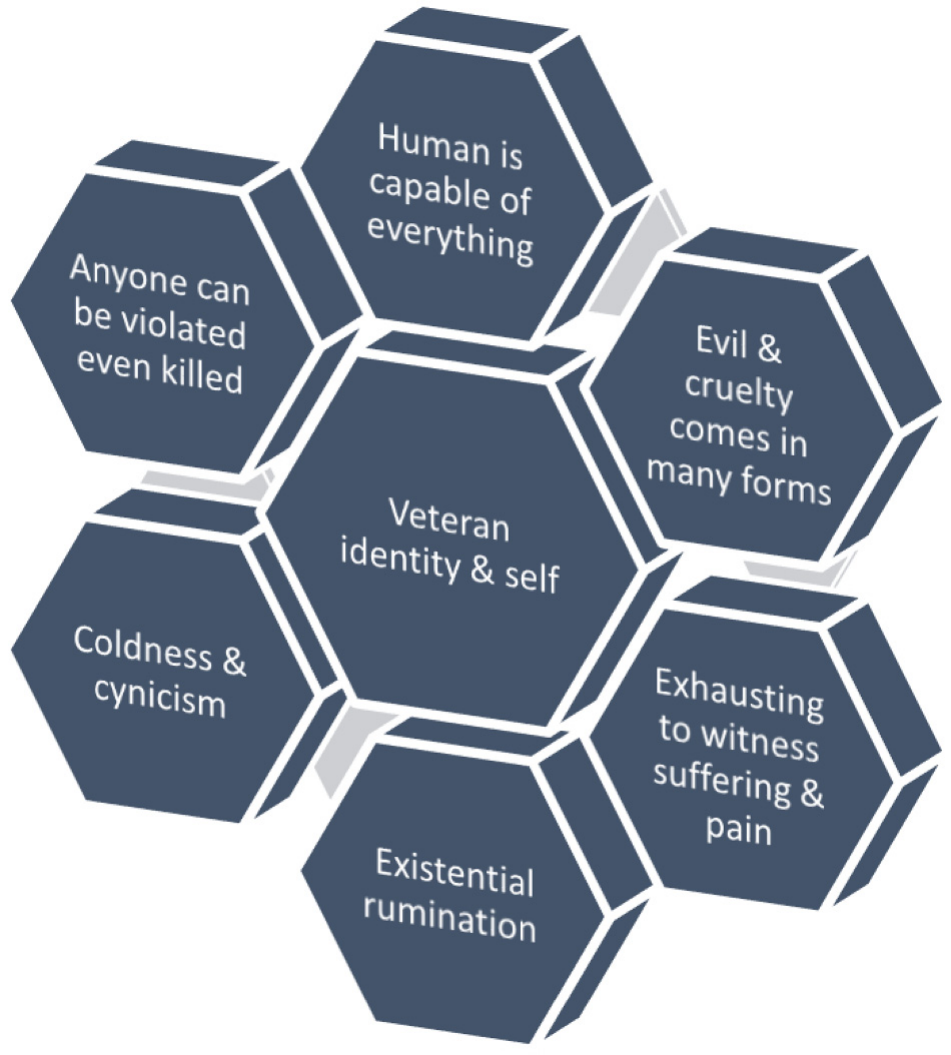
Constructions of evil on emerging spiritual veteran- and self-identity.

The experiences of evil within the whole interview sample in this study have led to six salient constructions of evil as they apply to one's emerging spiritual identity as a veteran and on the emerging spiritual self, as illustrated by the five cases and presented in Figure 2, below. The six implications can be seen as contours or elements that are more or less pronounced in the experiences and understandings of a veteran. Each and every element is not necessarily present within one and the same person, but several elements may co-exist.

The above constructions of evil are understood to be a common existential process amongst veterans in general one that shapes their emerging spiritual veteran identities, views of life, and eventually other identities due to their experiences during deployment in conflict and war zones. This may have an impact upon their mental health, but is more a matter of an existential journey open to interpretation and discussion. An existential journey, by its very core, is not a condition in need of medication and therapy. It is about life calling for an answer—a call which any person must answer (Tillich, 2014).

Each of these constructions represents empirical themes extracted from the analysis and understood in resonance to the working definition of evil suggesting that the existence of evil is necessary for informed and meaningful action and spiritual growth (Hick, 1966). The constructions actualize specific aspects of the problem of evil–not yet exhaustive but representative of this Swedish sample–as analytical starting points to discuss and unfold potential growth and development. For instance, “human is capable of anything” suggests an understanding that everything can happen and that veterans may have acted or will act in order to prevent such developments. “Anyone can be violated” suggests that one must protect what is considered sacred in life. These two themes together also suggest that veterans may have to check themselves to maintain spiritual integrity. “Evil and cruelty comes in many forms” points to the need to acknowledge evil in all its forms based on previous experiences. “Coldness/cynicism” suggest that something profound (e.g., the ability to love, trust, be open) can get lost in the encounter with evil. This potential loss can be met partly by reflection on the effects of evil and partly by informed and conscious (counter) action to re-cultivate the ability to love and trust. Moreover, it is “exhausting to witness suffering/pain” and this exhaustion of a veteran need additional strengthening experiences which fills the individual with joy, trust, love, warmth and care. Finally, “existential rumination” can stem from the exposure of evil in the world. It can be helpful to allow oneself to discuss such existential matters, including other potential constructions of evil as avenues for departure.

This actualizes pastoral care and counseling as a potential practice and approach to understanding the constructions of evil formed by veterans who then feel a need to vent and discuss their thoughts and experiences with an existential sparring partner; i.e., a pastoral care giver/counselor. Pastoral care is particularly applicable to the existential life–question–complex that veterans may want to unpack and discuss (Capps, 2001; Grimell, 2018c, 2022; Stallinga, 2013). Such an existential process is not about therapy or treatment, but about having the opportunity to vent and reflect upon concerns that must be considered as a natural and integral part of deployment to war zones (Grimell, 2020b, 2020c; Stallinga, 2013). The pastoral care giver in a Swedish context—in particular the military chaplain—is an existential expert trained and experienced in talking about difficult life-issues such as loss, pain, grief, meaning, the sacred, the good, the evil, and spiritual growth. The background of a military chaplain may differ across the globe. Swedish military chaplains have at least four years of academic theological education plus one additional years of pastoral training before ordination. As chaplains, additional courses to develop the toolbox are offered on a regular basis by the Church of Sweden and the armed forces. Military chaplains are also culturally competent in military contexts and a significant part of this group in the Swedish context are also deployed veterans (Grimell, 2020b, 2020c). However, it is important to stress the importance of employing caution when opening such themes together with confidants as it can potentially cause harm if the proper education, training, and experience are missing.

In addition, pastoral caregivers can guide veterans especially well beyond the mere recognition of evil and support conversation that patients bring onto ontological terrain and/or toward other emerging spiritual and identity themes. A consistent observation in this study has been that no interviewee has gone beyond recognition of the existence of evil. This is, of course, mainly because ontology was not explicitly addressed during the interviews by the researcher, which is a shortcoming of the study. But it may also be the case that the interviewees have not wanted to, or have not had the opportunity to, reason ontologically. A pastoral care giver has both an ontological language (Capps, 1995, 2001; Capps & Carlin, 2010) and a biblical tradition which, for example, embraces the complexities of war and life as well as biblical combat veteran types (Grimell, 2018b, 2018c, 2020d, 2020e). The absence of such existential expertise in the everyday life is especially salient in secular contexts. This suggests that veterans may have difficulties to finding people within their own social sphere to discuss these topics prior to and after deployments.

Deployed veterans have a certain understanding and openness to darker and brighter dimensions of life, given their experiences of evil, destruction, violence, and suffering—as well as community, loyalty, strong bonds to battle buddies, love, and self-sacrifice. The result of the encounter with good and evil during deployments is spiritual growth and an emerging spiritual veteran identity. This stands in stark contrast to civilians which have not been deployed to conflict zones to encounter “evil” as well as “the good” manifested through strong bonds to battle buddies, strong loyalty, and self-sacrifice.

These constructions of evil should not be confused with the concepts of “moral injury” (Litz et al., 2009; Shay, 2002, 2003) and “spiritual injury” (Berg, 2011). Moral injury can be used to describe a type of permanent moral issue, which impacts the mental health and well-being of veterans and can co-exist with a clinical PTSD diagnosis (Barr et al., 2022; Grimell, 2022). Moral injury is a type of potential (not yet clinically-recognized) diagnosis for understanding how actions that transgress deeply-held moral beliefs, or morally-charged identities, may have a profound impact on veteran's mental health (Atuel et al., 2021; Grimell, 2022). The evil in the model of constructions of evil on emerging spiritual veteran- and self-identity can be considered as a prerequisite or a support for spiritual growth. In Moral or Spiritual Injury there is instead an injury that does not necessarily help to foster growth.

There is also a potential risk that veterans can get stuck in a cold and cynical view of others, and life, which offers little room to trust that inviolable and sacred aspects of life can exist at some level in spite of the existence of evil. While veterans ruminate upon evil, a pastoral caregiver or military chaplain can work as an existential conversation partner, and can assist veterans in thinking further about the six constructions of evil uncovered in this study. More research into evil, from various religious traditions, in relation to veterans’ experiences amid war zone deployments—and their own processing of it and how chaplains can support them—is important and encouraged.

## Supplemental Material

sj-docx-1-pcc-10.1177_15423050231213418 - Supplemental material for Evil, Constructed: A Salient Part of an Emerging Spiritual Veteran Identity Click here for additional data file. Supplemental material, sj-docx-1-pcc-10.1177_15423050231213418 for Evil, Constructed: A Salient Part of an Emerging Spiritual Veteran Identity by Jan Grimell in Journal of Pastoral Care & Counseling

## Notes

1. Each participant was encouraged to suggest their pseudonym and LOLULL write his in capital letters which is the reason why it is capitalized.
